# CRISPRi screening reveals regulators of tau pathology shared between exosomal and vesicle-free tau

**DOI:** 10.26508/lsa.202201689

**Published:** 2022-10-31

**Authors:** Juan Carlos Polanco, Yevhen Akimov, Avinash Fernandes, Adam Briner, Gabriel Rhys Hand, Marloes van Roijen, Giuseppe Balistreri, Jürgen Götz

**Affiliations:** 1 Clem Jones Centre for Ageing Dementia Research, Queensland Brain Institute, The University of Queensland, Brisbane, Australia; 2 Institute for Molecular Medicine Finland, HiLIFE, University of Helsinki, Helsinki, Finland; 3 Faculty of Biological and Environmental Sciences, Molecular and Integrative Biosciences Research Program, University of Helsinki, Helsinki, Finland; 4 New South Wales Brain Bank, The University of Sydney, Sydney, Australia

## Abstract

The aggregation of the microtubule-associated protein tau is a defining feature of Alzheimer’s disease and other tauopathies. Tau pathology is believed to be driven by free tau aggregates and tau carried within exosome-like extracellular vesicles, both of which propagate trans-synaptically and induce tau pathology in recipient neurons by a corrupting process of seeding. Here, we performed a genome-wide CRISPRi screen in tau biosensor cells and identified cellular regulators shared by both mechanisms of tau seeding. We identified ANKLE2, BANF1, NUSAP1, EIF1AD, and VPS18 as the top validated regulators that restrict tau aggregation initiated by both exosomal and vesicle-free tau seeds. None of our validated hits affected the uptake of either form of tau seeds, supporting the notion that they operate through a cell-autonomous mechanism downstream of the seed uptake. Lastly, validation studies with human brain tissue also revealed that several of the identified protein hits are down-regulated in the brains of Alzheimer’s patients, suggesting that their decreased activity may be required for the emergence or progression of tau pathology in the human brain.

## Abstract (precis)

Using a genome-wide CRISPRi screen, we identified ANKLE2, BANF1, NUSAP1, EIF1AD, and VPS18 as novel regulators of tau pathology induced by both exosome-like extracellular vesicles and free tau seeds.

## Introduction

Tauopathies are neurodegenerative diseases in which the microtubule-associated protein tau undergoes a process of aggregation and fibrillization that gives rise to the pathological hallmark known as neurofibrillary tangles (Polanco et al, 2018b; Chung et al, 2021). Alzheimer’s disease (AD) is a secondary tauopathy, in which amyloid plaques feature as an additional histopathological feature, a characteristic that is lacking in primary tauopathies such as frontotemporal lobar degeneration with tau (FTLD-tau), argyrophilic grain disease, progressive supranuclear palsy, or corticobasal degeneration (Polanco et al, 2018b; Chung et al, 2021). AD accounts for up to 80% of all dementia cases worldwide and currently affects more than 50 million people (Polanco et al, 2018b). The pathological aggregation of tau impairs neuronal physiology at diverse levels, including axonal transport, action potential firing, synaptic plasticity, nuclear transport, chromatin structure, and mitochondrial function, which together lead to neurodegeneration and the ensuing cognitive and behavioral impairment (Eckert et al, 2014; Polanco et al, 2018b; Hill et al, 2020). A characteristic of tau aggregates, which are composed of misfolded tau modified by hyperphosphorylation and other post-translational modifications, is that they form proteopathic seeds that template the misfolding of physiological tau, and induce it to also misfold and form oligomers and fibrils (Polanco & Götz, 2015; Jucker & Walker, 2018). This process is not restricted to the neurons in which the seeds form, but these can propagate trans-synaptically and then be taken up by recipient neurons where they cause pathology by corrupting the native conformation of soluble tau (Polanco & Götz, 2021). This propagation requires release by donor cells and subsequent internalization by recipient cells, which is brought about primarily via endocytosis. Importantly, to corrupt the conformation of physiological endogenous tau, the endocytosed tau seeds need to escape from the endolysosomes into the cytosol (Flavin et al, 2017; Chen et al, 2019; Polanco et al, 2021).

Several studies have revealed that the propagation of tau seeds can be potentially controlled by interfering at multiple steps such as the production of the different tau seeds, and their neuron-to-neuron transmission, internalization, endosomal escape into the cytosol, and cytoplasmic autophagy of newly forming tau aggregates (Polanco & Götz, 2021). Fundamentally, two forms of tau seeds have been demonstrated to induce tau aggregation: (i) naked, that is, vesicle-free tau in the form of oligomers or fibrils (Holmes et al, 2014; Calafate et al, 2015); and (ii) tau encapsulated by the membranes of exosome-like extracellular vesicles (Polanco et al, 2016, 2018a; Miyoshi et al, 2021; Ruan et al, 2021; Leroux et al, 2022; Saroja et al, 2022), hereafter referred to as exosome-like EVs or exosomal tau seeds. There is an ongoing debate about which type of tau seed is critical in the progress of tau pathology. One recent report, which used preparations from the same human AD brain tissue, claimed that exosome-like EVs have higher transmissibility and cause a more potent induction of tau pathology than vesicle-free tau seeds, whether oligomeric or fibrillar (Ruan et al, 2021). However, any potential therapeutic approach that only targets either exosome-like EVs or free aggregates would confer incomplete protection from the tau pathology induced by the non-targeted seeds (Polanco & Götz, 2021). Therefore, it is crucial to identify the regulators of tau pathology that control both forms of tau seeding.

Here, we report novel regulators of seeded tau aggregation, which we discovered using a genome-wide CRISPR interference (CRISPRi) genetic screen (Sanson et al, 2018) coupled to a human cellular model of tau aggregation known as tau biosensor cells (Holmes et al, 2014) engineered to report tau aggregation by generating a FRET signal. Downstream bioinformatic analyses subsequently identified multiple hits, several of which we functionally validated, revealing that their individual knockdown predisposed the cells to tau aggregation. Interestingly, we found that several targets were also down-regulated in the brains of AD patients, suggesting that their decreased activity may be required for the emergence or progression of tau pathology in the human brain.

## Results

### Novel cellular regulators of tau pathology revealed by genome-wide CRISPRi screening

**Figure 1.**
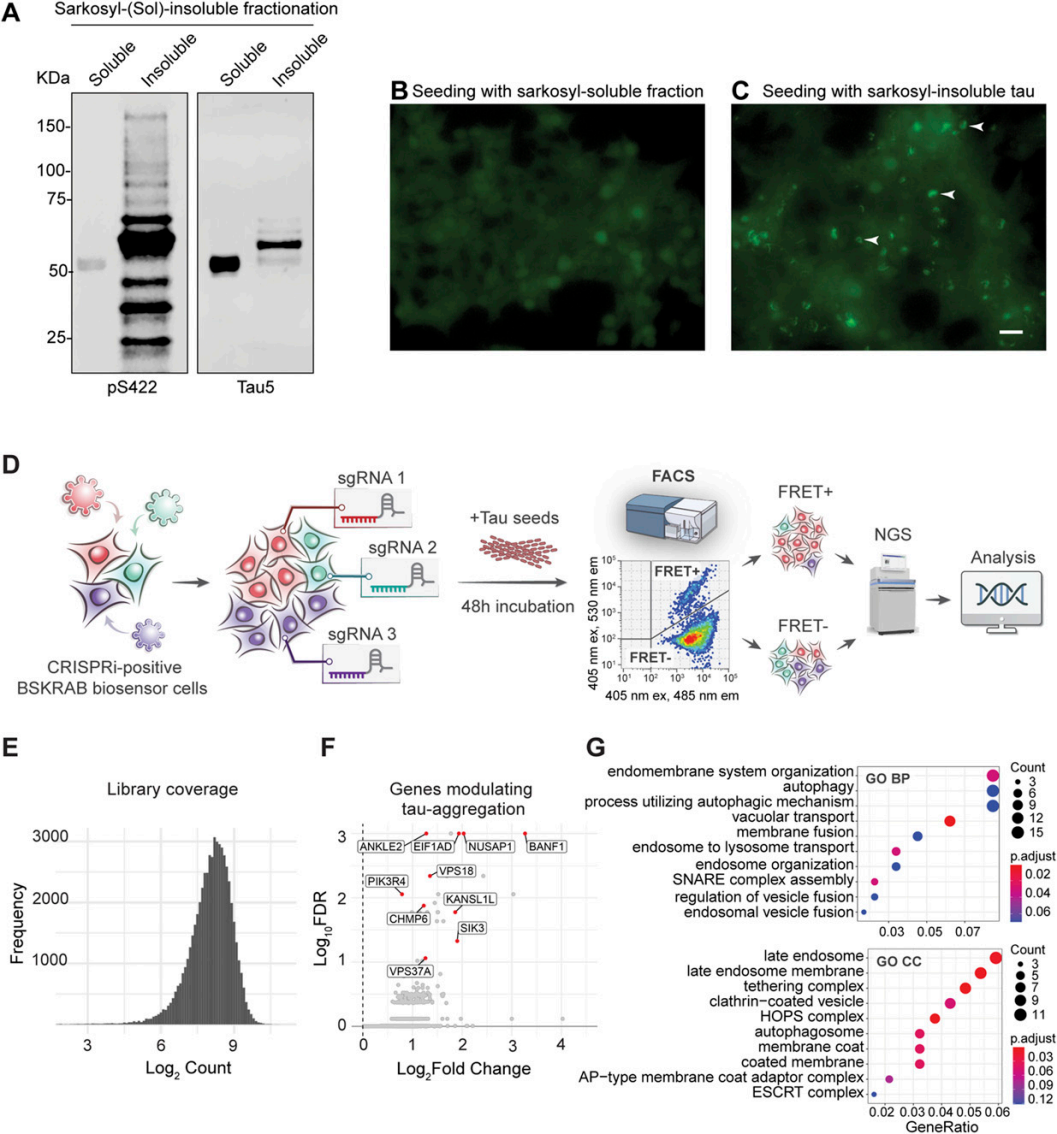
Unbiased discovery of regulators of tau aggregation using pooled CRISPRi screens and sarkosyl-insoluble tau. **(A)** Western blot analysis of sarkosyl-soluble and sarkosyl-insoluble fractions obtained from brains of P301L tau transgenic rTg4510 mice. The insoluble fraction exhibits phosphorylated tau (pS422) and high molecular weight total tau (Tau5). **(B, C)** Epifluorescence microscopy detecting tau RD-YFP in tau biosensor cells 48 h after treatment with sarkosyl-soluble (B) and sarkosyl-insoluble tau (C). Brighter spots (arrowheads) representing tau aggregates only appear in cells that were treated with sarkosyl-insoluble tau. Scale bar: 50 µm. **(D)** Schematic representation of the pooled CRISPRi screen. Tau biosensor cells expressing the FRET pair tau RD-CFP and tau RD-YFP together with lentiviral KRAB-dCas9 (BSKRAB) were transduced with the Dolcetto CRISPRi library containing pooled lentiviral sgRNAs targeting ∼18,000 genes, followed by incubation with sarkosyl-insoluble tau (vesicle-free tau seeds). 48 h later, the cells were sorted into FRET(+) and FRET(−) populations using FACS. Samples were processed to generate an NGS sequencing library and sequenced on a NextSeq 500 instrument. **(E)** Frequency distribution of mean sgRNA read counts across all samples. 39 out of 57,050 sgRNAs (0.068%) were not detected. **(F)** Volcano plot showing genewise log_2_ fold changes of sgRNA counts versus false discovery rate. False discovery rate values were based on robust ranking aggregation from MAGeCK (Li et al, 2014). Genes that were followed up are highlighted in red. **(G)** Gene ontology-term enrichment analysis of the top 200 positive regulators of tau aggregation identified by CRISPRi screening and enrichment for relevant pathways.

Tau biosensor cells (Holmes et al, 2014), designed to fluorescently display the extent of induced tau aggregation, have been widely used to study tau propagation and aggregation (Sanders et al, 2014; Mirbaha et al, 2015; Briner et al, 2020; Polanco et al, 2021). Moreover, we have previously shown that tau biosensor cells internalize both exosome-like EVs and vesicle-free tau isolated from the brains of rTg4510 tau transgenic mice, resulting in the formation of intracellular tau inclusions (Polanco et al, 2016). To screen for novel regulators of tau pathology, we coupled tau biosensor cells to an optimized genome-wide CRISPRi library (Sanson et al, 2018) (Fig 1). First, we transduced these cells with a lentiviral KRAB-dCas9 construct, which expresses a nuclease-dead Cas9 (dCas9) fused to the transcription-repressing KRAB domain to efficiently elicit gene silencing (Qi et al, 2013; Sanson et al, 2018). These modified tau biosensor cells (hereafter named BSKRAB cells) were then transduced with lentiviruses comprising the pooled whole-genome Dolcetto sgRNA CRISPRi library (Sanson et al, 2018), and subsequently treated with sarkosyl-insoluble tau isolated from rTg4510 tau transgenic mouse brains (Santacruz et al, 2005). We chose this form of vesicle-free tau seeds, which contain post-translational modifications such as phosphorylation (Fig 1A), as previous studies have shown that sarkosyl-insoluble brain-derived aggregates are more potent seeders than aggregates generated in vitro with recombinant tau (Falcon et al, 2015). Indeed, 48 h after treatment of BSKRAB cells with sarkosyl-insoluble tau, we could detect robust seeded tau aggregation (Fig 1C), whereas the sarkosyl-soluble fraction did not induce tau aggregates (Fig 1B). Importantly, all seeding assays were performed without Lipofectamine, an agent that strongly increases seeded tau aggregation (Polanco et al, 2016) but bypasses physiological vesicular trafficking by inducing and enhancing endolysosomal permeabilization (Polanco et al, 2021), which could create false-positive results in a genetic screen that uses tau aggregation as a readout.

Using FACS, we obtained two cell populations: FRET-positive cells harboring induced tau aggregates and FRET-negative cells without aggregates. Genomic DNA was isolated, followed by next-generation sequencing (NGS) to quantify which sgRNAs were enriched in the FRET-positive cells using the MAGeCK bioinformatic pipeline (Li et al, 2014) (Fig 1D). Analysis of the library coverage showed that a large majority of sgRNAs were detected (Fig 1E). Our analysis revealed 23 genes (false discovery rate, FDR, < 5%; Table S1) that were positively enriched in FRET-positive cells with tau aggregation (Fig 1F). Two of our 23 top hits, CHMP6 and VPS13A (FDR < 5%; Table S1), had been previously reported as regulators of endosomal integrity during tau aggregation (Chen et al, 2019), whereas BANF1 (FDR < 5%; Table S1) was recently identified in a CRISPR knockout screen with tau biosensor cells from an independent group (Prissette et al, 2022). Furthermore, when the first 200 top gene hits, selected based on robust ranking aggregation from MAGeCK (Li et al, 2014), were analyzed for pathway and protein complex enrichment using GO-BP (gene ontology-biological process) and GO-CC (gene ontology-cellular component) annotations, they were found to be primarily enriched in pathways of the late endosome, autophagosome, and tethering complexes coordinating endosome and lysosome fusion, suggesting an increase in tau aggregation due to a loss of function of genes with a role in autophagy and late endosomes (Fig 1G).

Table S1 List of top hits with FDR < 25%.

### Validation of CRISPRi hits reveals genes that predispose cells to both vesicle-free and exosomal seeded tau aggregation

**Figure 2.**
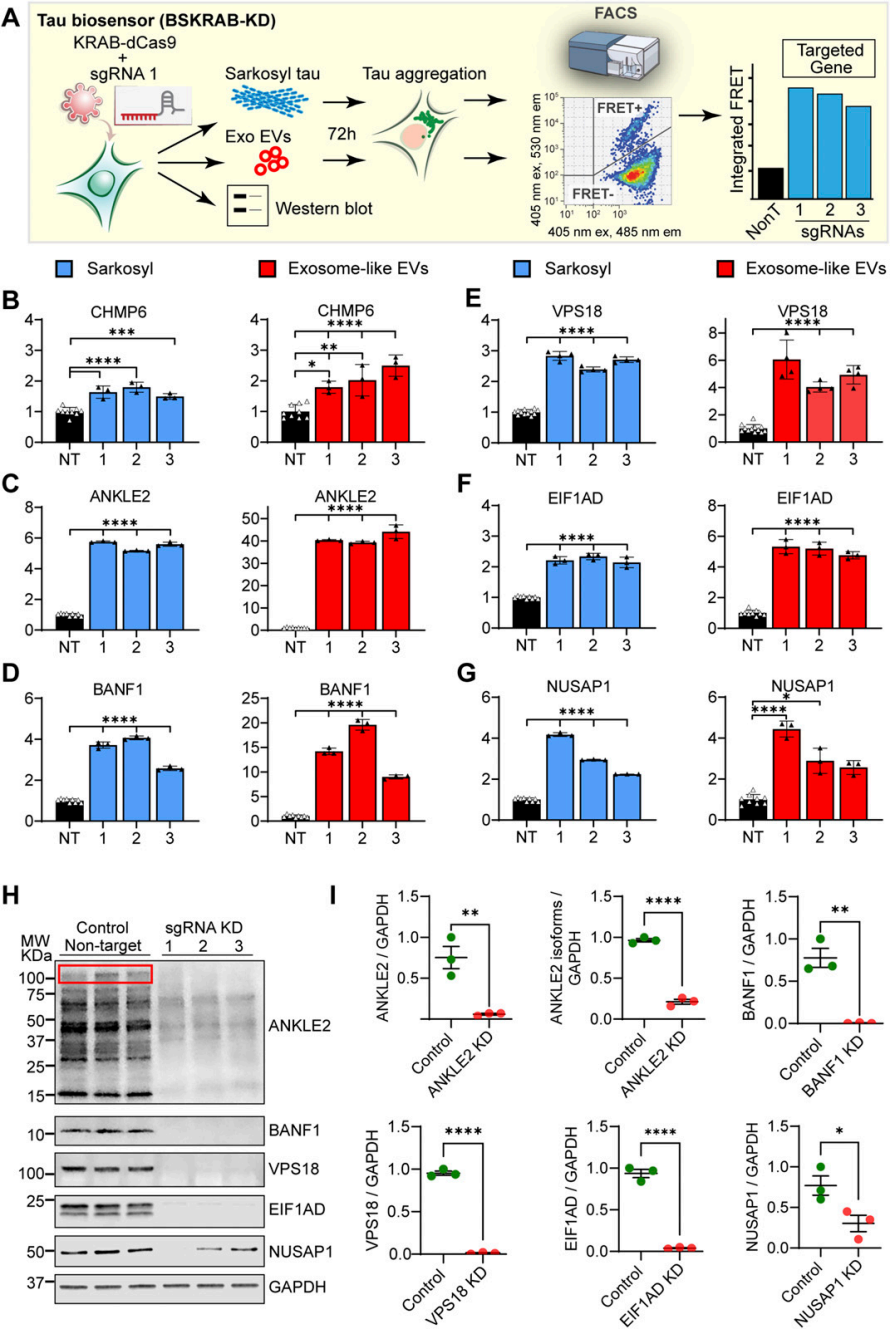
Functional validation of bioinformatic hits using individual CRISPRi knockdowns followed by incubation with exosome-like EVs and vesicle-free tau seeds. **(A)** Schematic representation of the workflow for functional validation. Individual sgRNAs were used to silence the corresponding genes in combination with KRAB-dCas9 in tau biosensor cells (BSKRAB-KD), then treated with either sarkosyl-insoluble tau (vesicle-free tau seeds) or exosome-like EVs for 72 h, followed by detection and quantification of tau aggregation using FRET flow cytometry. A fraction of the same BSKRAB-KD cells was also grown for 72 h to corroborate the knockdown of protein expression using Western blots. **(B, C, D, E, F, G)** Integrated FRET intensities represent levels of tau aggregation upon knocking down the different targets. The control black column (NT) is the average obtained with three independent non-targeting sgRNAs (n = 3) assessed in triplicate. Control cells were compared with knockdown cells targeted individually (1, 2, and 3). Error bars represent the SEM for n = 3, *P < 0.05; **P < 0.01; ***P < 0.001; and ****P < 0.0001. Each single targeting sgRNA increased tau aggregation with both exosomal and vesicle-free tau seeds. **(C, D)** Interestingly, ANKLE2 and BANF1 (C, D) appear to induce a stronger effect on tau aggregation induced by exosome-like EVs. **(H)** Quantitative Western blot analysis of BSKRAB-KD knockdown cells. Each sgRNA generated a protein knockdown of the targeted gene. Note that the ANKLE2-specific antibody reacted with several isoforms, including the canonical variant sized 104–117 kD (red box outline); however, all isoforms were down-regulated when the ANKLE2 locus was silenced. Similarly, the EIF1AD antibody recognized the canonical isoform of 19 kD and one additional variant of lower molecular weight, both being silenced with the individual sgRNA against EIF1AD. **(I)** Quantification of the extent of protein knockdown for the different targeted genes. Error bars represent the SEM for n = 3, *P < 0.05; **P < 0.01; and ****P < 0.0001.

Bioinformatic analysis and calculations of FDR values allow for determining the probability that a gene is a false positive (Table S1). The arbitrary threshold of FDR < 5% is generally well accepted, but even under this threshold, some genes could be false positives, and it is also known that additional true hits can be identified with FDR > 5%, highlighting the importance of functional validation. Therefore, we next established a pipeline for functional validation and, as a proof of principle, functionally validated 10 of our top hits (Fig 1F), which were selected considering that they have been shown to be expressed in neurons of the human brain and that these genes covered top (BANF1, EIF1AD, NUSAP1, and ANKLE2), middle (VSP18, PIK3R4, CHMP6, and KANSL1L), and lower (SIK3 and VPS37A) FDR values. Each gene was individually targeted with three sgRNAs from the Dolcetto library to achieve CRISPRi-mediated gene silencing in tau biosensor cells (hereafter named BSKRAB-KD), using a lentiviral vector in which both KRAB-dCas9 and the individual sgRNA were contained within a single construct. BSKRAB-KD cells in which individual genes had been silenced were then compared with a control that was obtained using the mean of three non-targeting sgRNAs (Fig 2A).

**Figure 3.**
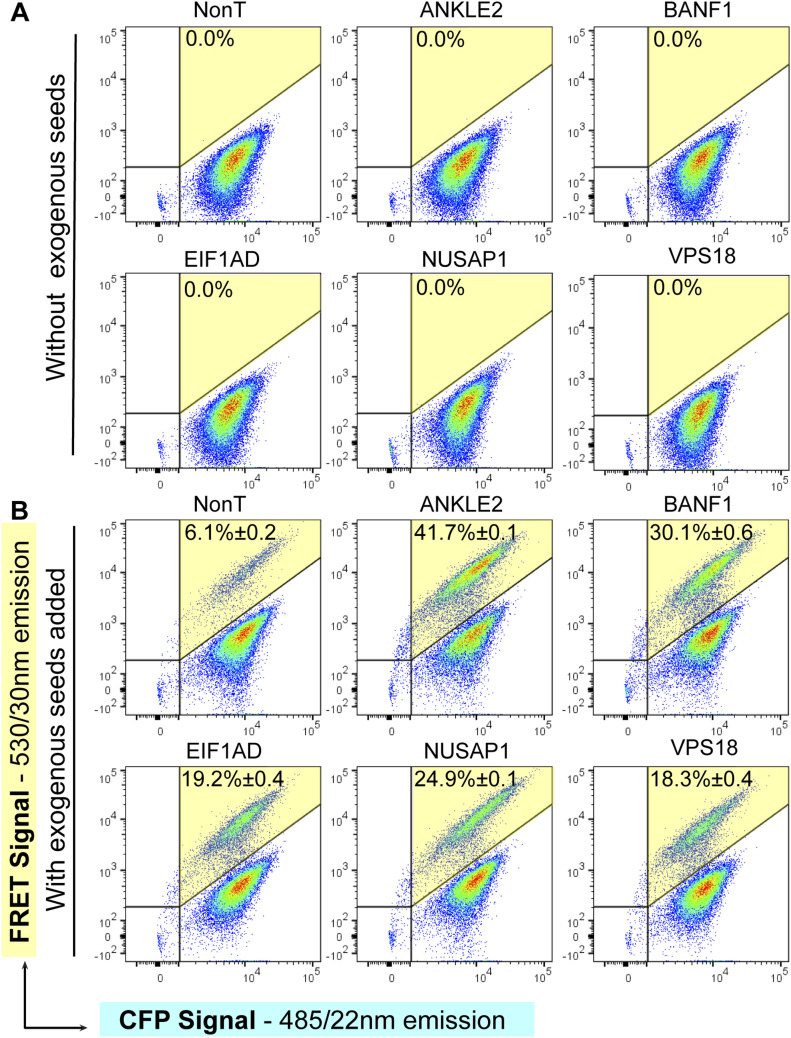
Individual knockdowns of ANKLE2, BANF1, VPS18, EIF1AD, or NUSAP1 do not induce spontaneous tau aggregation. **(A)** FACS plots of knockdown and control cells (non-targeting sgRNA) without adding exogenous tau seeds. Quadrants (Q2 shaded in yellow) in which FRET-positive cells were detected revealed the absence of a FRET signal in both control (NonT) and knockdown cells for ANKLE2, BANF1, VPS18, EIF1AD, and NUSAP1, indicating that no spontaneous tau aggregation was initiated. **(B)** However, these cells (bottom panels) showed FRET-positive cells only after tau seeds (400 ng of sarkosyl tau) were added, implying the requirement for an exogenous tau seed to trigger the aggregation of endogenous tau. Percentages of FRET-positive cells in Q2 are shown (n = 3, average ± SEM, 40,000 cells/experiment were analyzed).

**Figure S1.**
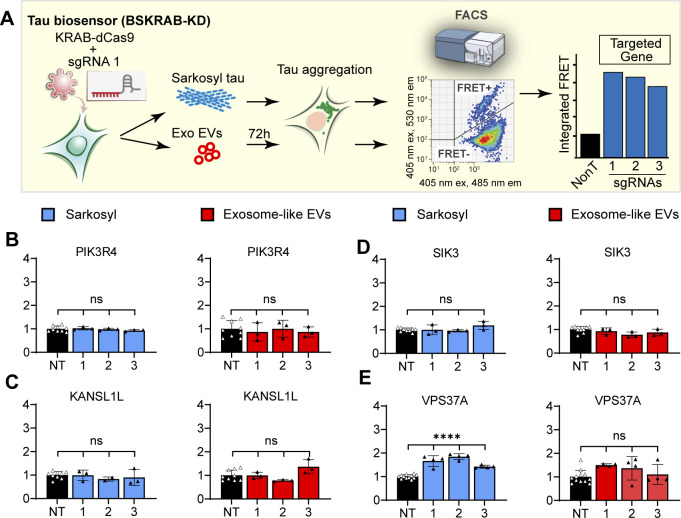
Additional bioinformatic hits that were functionally validated using individual CRISPRi knockdowns. **(A)** Workflow for functional validation. Individual sgRNAs were used to silence the targeted gene in combination with KRAB-dCas9 in tau biosensor cells (BSKRAB-KD), then treated with either sarkosyl-insoluble tau or exosomal tau seeds for 72 h, followed by detection and quantification of tau aggregation using FRET flow cytometry. **(B, C, D, E)** Integrated FRET intensities represent levels of tau aggregation under knockdown conditions for the different gene hits. The control black column (NT) is the average obtained with three independent non-targeting sgRNAs (n = 3) assessed in triplicate. Control cells were compared with knockdown cells targeted individually (1, 2, and 3). Individual silencing of PIK3R4, SIK3, and KANSL1L did not result in a significant increase in tau aggregation. Knockdown of VPS37A produced a weak potentiation of tau aggregation, in particular for tau in exosome-like EVs. Error bars represent SEM for n = 3; ****P < 0.0001; ns, not significant.

For validation, the cells were treated with either vesicle-free tau seeds (sarkosyl-insoluble tau) or exosome-like EVs isolated from rTg4510 mouse brains, followed by detection and quantification of tau aggregation using FRET flow cytometry (Fig 2A). We used one of the 10 hits, CHMP6, for benchmarking purposes (Fig 2B), given that its knockdown has previously been reported to induce tau aggregation and it has been extensively characterized (Chen et al, 2019). We found that apart from the CHMP6 benchmark (Fig 2B), the individual knockdowns of ANKLE2, BANF1, NUSAP1, EIF1AD, and VPS18 strongly and significantly promoted tau aggregation induced by both exosome-like EVs and vesicle-free tau seeds (Fig 2C–G). Remarkably, ANKLE2 and BANF1 showed a substantially stronger effect on tau aggregation induced by exosome-like EVs (Fig 2C and D). Of note, silencing of ANKLE2 resulted in a 40-fold induction of seeding when treated with exosome-like EVs, compared with a sixfold induction when treated with vesicle-free tau (Fig 2C). Furthermore, when whole-cell lysates from the five types of knockdown cells were analyzed by quantitative Western blotting (Fig 2H), we found that each of the sgRNAs was able to induce gene silencing of the targeted genes (Fig 2I), demonstrating the reproducibility and robustness of CRISPRi-mediated gene silencing. Similarly, the fact that three different silencing sgRNAs strongly reduced the expected bands on Western blots supports the specificity of the commercial antibodies used in our study. Of note, we found that the polyclonal ANKLE2 antibody reacted with the canonical 104–117 kD isoform (Fig 2H, red box outline) and other isoforms of lower molecular weight, which were also down-regulated when the ANKLE2 locus was silenced (Fig 2H). We next showed that the knockdown of ANKLE2, BANF1, VPS18, EIF1AD, or NUSAP1 did not induce spontaneous tau aggregation and that increases in FRET-positive cells were only observed when mutant cells were treated with exogenous tau seeds (Fig 3). This indicates that these genes regulate seeded tau aggregation and not the spontaneous aggregation of tau. We also evaluated PIK3R4, KANSL1L, and SIK3, but these hits did not potentiate tau aggregation in the validation assays (Fig S1) and are likely false positives. VPS37A exhibited a low potentiation and was therefore not followed up (Fig S1).

### Increased tau aggregation is not linked to mechanisms that affect tau uptake

**Figure 4.**
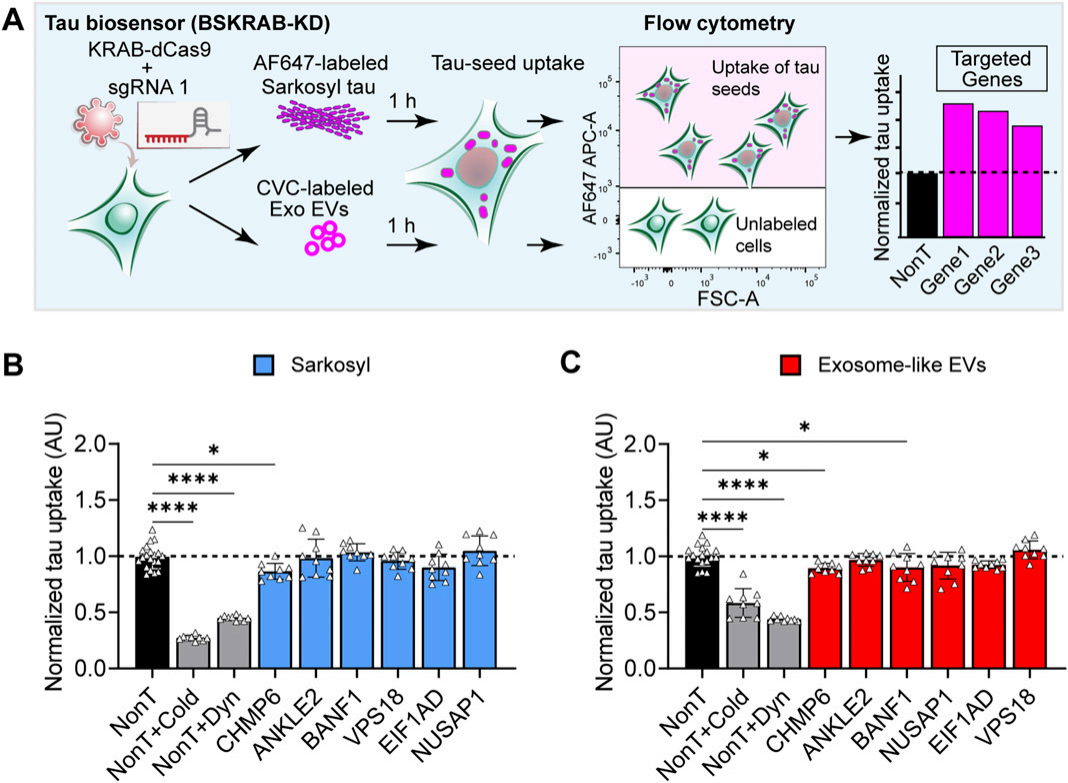
Analysis of the levels of tau seed uptake for the different knockdown cells. **(A)** Schematic of the workflow for quantifying the uptake of exosomal and vesicle-free tau seeds. BSKRAB-KD knockdown cells were treated with far-red–labeled tau seeds, either Alexa Fluor 647–labeled sarkosyl tau or CVC-labeled exosome-like EVs for 1 h, followed by the detection of cells harboring the labeled tau seeds using flow cytometry. Tau uptake was quantified by measuring the mean fluorescence intensity in far-red–positive cells. **(B, C)** None of the gene knockdowns generated a significant increase in tau seed uptake (dashed line), suggesting that these genes are downstream of tau seed internalization and therefore impact tau aggregation through cell-autonomous mechanisms. Three different non-targeting sgRNAs (n = 3) assayed in triplicate were used as control (black column) and compared with three knockdown sgRNAs targeting each gene, which were pooled for comparison. As for the control, uptake inhibition controls were completed using three independent non-targeting sgRNAs (grey columns; Dyn, dynamin inhibitor; and cold, incubation at 4°C). Error bars represent the SEM for n = 9, *P < 0.05 and ****P < 0.0001.

Having demonstrated that the individual gene knockdowns of ANKLE2, BANF1, NUSAP1, EIF1AD, and VPS18 resulted in enhanced seeded tau aggregation (Fig 2), we asked whether these increases in tau aggregation could be merely the result of a higher uptake of the tau seeds. To assess the level of internalization of tau seeds, we labeled sarkosyl-insoluble tau with the far-red dye Alexa Fluor 647, whereas the membranes of exosome-like EVs were labeled with the far-red fluorescent membrane probe CellVue Claret (CVC). We then measured the level of uptake in individual gene knockdowns of BSKRAB-KD cells using flow cytometry (Fig 4A). Our results revealed that none of the gene knockdowns impacted cellular uptake as shown for vesicle-free tau (Fig 4B) and exosome-like EVs (Fig 4C), indicating that the observed increases in tau aggregation (Fig 2) were not due to the increased internalization but rather due to a cell-autonomous mechanism downstream of the seed uptake. In line with widely accepted procedures, uptake inhibition using either a potent inhibitor of dynamin-dependent endocytosis or incubation at a low temperature (4°C) was used to validate these uptake assays and to further demonstrate that the labeled seeds were internalized and not simply tethered to the membrane surface (Fig 4B and C).

### Decreased levels of VPS18, NUSAP1, and EIF1AD are found in the brains of Alzheimer’s patients

**Figure 5.**
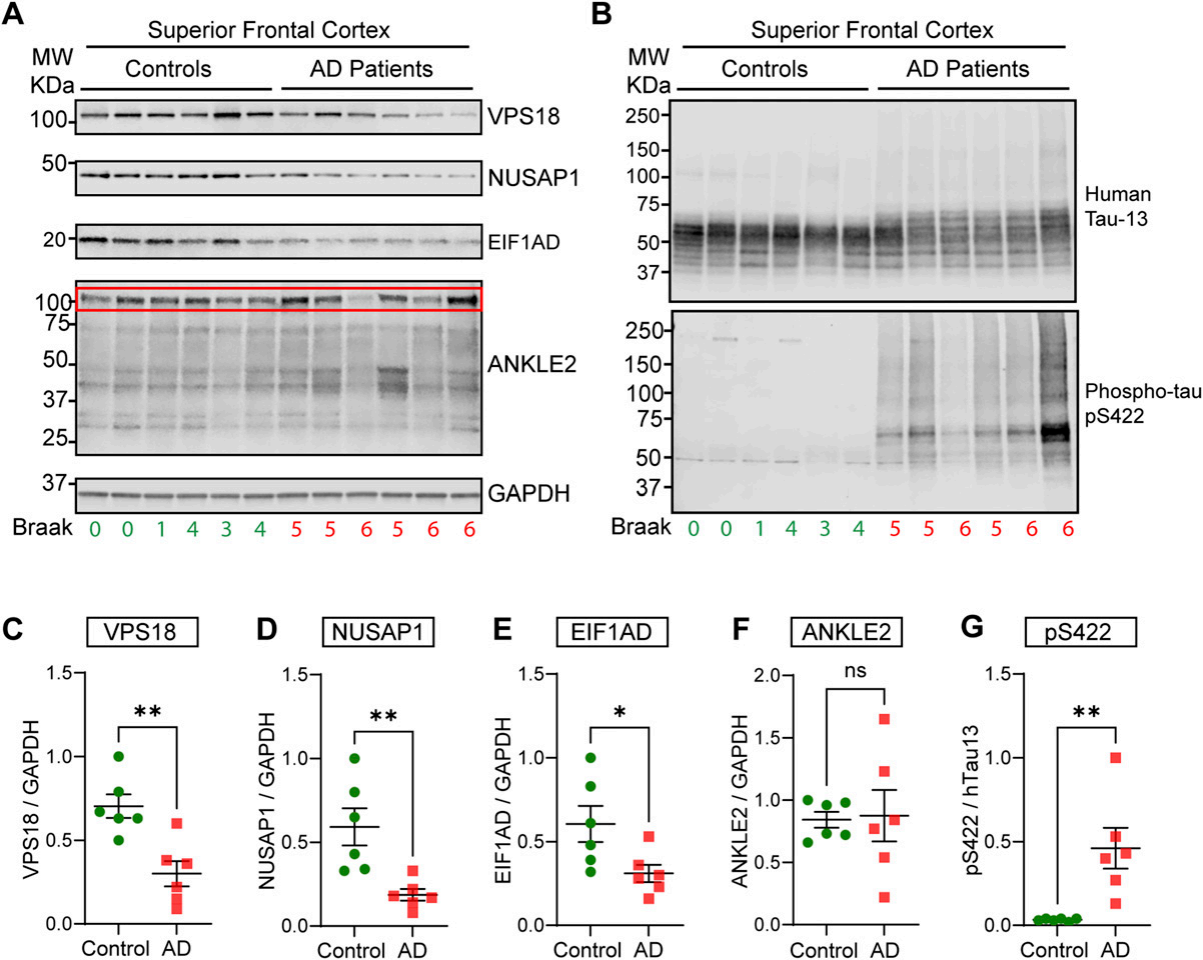
Quantitative Western blot analysis of novel validated regulators in postmortem brain tissue from Alzheimer’s patients. **(A)** Western blot analysis of validated novel regulators using postmortem cortical brain samples (superior frontal cortex) from Alzheimer’s patients (AD). For the pathological data of the patients, see Table 1. Antibodies against human VSP18, NUSAP1, EIF1AD, and ANKLE2 were used. **(B)** Detection of human tau (Tau-13 antibody) and tau phosphorylated at Ser422 (pS422 antibody) in the human brain samples. **(C, D, E, F)** Quantification of the protein levels in control and AD samples revealed that VPS18 (C), NUSAP1 (D), and EIF1AD (E) are significantly down-regulated in AD patients. **(F)** However, ANKLE2 levels (F) were not statistically different. Of note, the canonical isoform with a size of 104–117 kD (red box outline) was the predominant isoform detected in human brains, and therefore, only this isoform was quantified. **(G)** Quantification of the ratio of pS422/hTau-13 revealed a substantial increase in tau phosphorylation at Ser422 in AD samples. Error bars represent the SEM for n = 6, *P < 0.05; **P < 0.01; and ns, not significant.

Validation studies in tau biosensor cells revealed that decreasing the levels of key cellular factors resulted in increased tau aggregation. To validate our hits further, we asked whether tau aggregation in AD patients correlates with the down-regulation of our functionally validated CRISPRi targets. To address this, we performed a quantitative Western blot analysis of postmortem brain samples from AD patients (Fig 5 and Table 1). Strengthening the relevance of our screen for the human condition, our analysis revealed that VPS18, NUSAP1, and EIF1AD were all down-regulated in cortical AD brain tissue (Fig 5A and C–E), which is characterized by a substantial accumulation of phosphorylated and aggregated tau (Fig 5B and G). Surprisingly, no statistically significant differences were found for ANKLE2 levels between AD and control patients (Fig 5F), although the data were heterogenous for the patients analyzed. Of note, only the predominant, canonical ANKLE2 104–117 kD isoform (Fig 5A, red box outline) was quantified (Fig 5F). We did not detect BANF1 via Western blotting in our cortical brain samples, possibly reflecting low expression levels. Together, our data suggest that the decreased activity of VPS18, NUSAP1, and EIF1AD in AD may be an upstream pathogenic event that enhances the aggregation and interneuronal spreading of pathological tau throughout the human brain.

**Table 1.** Human cases used for Western blot analysis of validated novel regulators using brain samples from the superior frontal cortex.

#	Function	Age (yr)	Gender	Disease duration (yr)	Braak stage	Postmortem delay (h)
1	Control	93	Female	0	0	21
2	Control	85	Female	0	0	23
3	Control	89	Female	0	1	23
4	Control	102	Female	0	4	5
5	Control	97	Female	0	3	16
6	Control	92	Female	0	4	5
7	Alzheimer’s disease	83	Female	7	5	3
8	Alzheimer’s disease	100	Female	17	5	4
9	Alzheimer’s disease	85	Female	5	6	10
10	Alzheimer’s disease	100	Female	11	5	3
11	Alzheimer’s disease	84	Female	13	6	6
12	Alzheimer’s disease	75	Female	8	6	14

## Discussion

Tau forms pathological aggregates in tauopathy but how these are generated is only incompletely understood. In the familial forms of the primary tauopathy FTLD-tau, the disease is caused by autosomal dominant mutations in the tau-encoding *MAPT* gene (Goedert & Jakes, 2005; Strang et al, 2019), demonstrating that tau dysfunction by itself can lead to neurodegeneration and dementia. The vast majority of tauopathies, however, are sporadic (Poorkaj et al, 2001). Here, we identified and functionally validated ANKLE2, BANF1, VPS18, NUSAP1, and EIF1AD as cellular regulators whose knockdown predisposes cells to tau aggregation. The products of these genes therefore function as host restriction factors that control tau pathology. Importantly, given that our hits did not affect the uptake of tau seeds, these genes are presumed to be downstream of tau internalization, causing tau aggregation via cell-autonomous mechanisms. Such cell-autonomous mechanisms appear to be exclusive of seeded tau aggregation, given that the knockdowns of the discovered regulators did not induce the spontaneous aggregation of tau in the reporter cell line, which highlights the requirement of an exogenous tau seed to trigger the misfolding of endogenous tau. Furthermore, we showed that VPS18, NUSAP1, and EIF1AD are all down-regulated in AD brain samples, supporting the notion that these regulators may be important for the development of tauopathy in the human brain.

Regarding risk genes, genome-wide association studies have been instrumental in identifying genes linked to an altered risk of developing tauopathies such as AD or FTLD-tau (Ferrari et al, 2014; Cuyvers & Sleegers, 2016; Gan et al, 2018). Paradoxically, when a total of 22 AD risk modifier genes revealed by genome-wide association studies were ablated in the cellular model of tau biosensor cells, the corresponding gene knockouts affected neither the uptake of tau seeds nor the development of tau aggregates, indicating that these risk genes may not be directly involved in tau propagation (Kolay & Diamond, 2020). In our study, we employed a functional approach using CRISPR-based genomics to identify genes that regulate seeded tau aggregation induced by both vesicle-free and exosomal tau seeds, with the goal of identifying disease-relevant genes and unveiling potential causal determinants in tauopathy.

Our analysis of the biological pathways in which the first 200 gene hits of our screen could be operating revealed that these genes are mostly involved in pathways of the late endosome, autophagosome, and tethering complexes that coordinate endosome and lysosome fusion. These results support the notion that decreased autophagy or impaired late-endosome activity increases tau aggregation, perhaps due to an inability to degrade newly forming tau aggregates. For instance, VPS18 and CHMP6 (FDR < 5%) were two validated positive hits that are involved in vesicle-mediated protein trafficking to lysosomal compartments, and sorting of endosomal cargo proteins into endosomal multivesicular bodies. Similarly, CHMP6 and VPS13A from our top list (FDR < 5%) were also identified in a previous report that used a CRISPRi library restricted to genes in the endosomal pathway (Chen et al, 2019). The fact that these genes were also found in our genome-wide screen (which was not limited to endosomal genes as in the study by Chen and colleagues) strengthens the confidence in the role of CHMP6 and VPS13A in regulating seeded tau aggregation. Interestingly, CHMP2B, a functional relative of CHMP6, has been linked to familial forms of FTLD-tau (Urwin et al, 2010; Clayton et al, 2015). Similarly, VPS18 was found to be down-regulated in human induced pluripotent cell-derived cerebral organoid models obtained from familial AD tissue (Zhao et al, 2020). VPS18 is a subunit of the mammalian homotypic fusion and vacuole protein sorting complex that regulates the fusion of endosomes and autophagosomes with lysosomes (Wartosch et al, 2015). Thus, our study supports previous investigations, which suggest that autophagic dysfunction and endolysosomal dysfunction are a driving force in the development of tauopathies and neurodegeneration more generally (Nixon, 2005; Nixon & Yang, 2011; Whyte et al, 2017; Gan et al, 2018; Malik et al, 2019; Polanco & Götz, 2021), highlighting the importance of endolysosomal dysfunction and autophagic dysfunction as a risk factor in neurodegenerative diseases.

Intriguingly, gene silencing of ANKLE2 and BANF1 resulted in some of the most potent effects on tau aggregation, yet little is known about the role of these genes in tauopathies. ANKLE2 has been found to be down-regulated in the transcriptomes from laser-captured CA1 neurons and microglia from AD brains, and in AD hippocampal homogenates (Mastroeni et al, 2017). However, we were unable to corroborate the down-regulation of ANKLE2 by Western blotting using cortical brain tissue from AD and control aging patients, possibly due to differences between hippocampal and cortical tissues. Although ANKLE2 was not down-regulated in our study, this does not rule out a potential loss of function of ANKLE2 because of an altered post-translational modification such as phosphorylation or acetylation (Kaufmann et al, 2016; Link et al, 2019), or a potential inactivation due to protein aggregation. We speculate that one mechanism by which ANKLE2 may influence tau aggregation is through its binding to PP2A (Asencio et al, 2012), a known protein serine/threonine phosphatase that directly regulates tau phosphorylation and physically binds to tau (Kins et al, 2001; Sontag et al, 2012), potentially acting together with PP2A to dephosphorylate tau and thereby reduce tau aggregation. Another mechanism by which ANKLE2 could affect tau pathology is through the regulation of the integrity of the nuclear envelope, which can be affected by ANKLE2-regulated phosphorylation of BANF1 (Asencio et al, 2012). It has been reported that reducing ANKLE2 levels disrupts the nuclear envelope and affects its morphology (Asencio et al, 2012; Link et al, 2019). Interestingly, anomalous invaginations of the nuclear envelope have been found in AD and FTLD-tau patients (Paonessa et al, 2019; Kang et al, 2021), and tau accumulates close to these invaginations (Paonessa et al, 2019), with evidence suggesting that liquid-liquid phase separation of tau at the nuclear envelope might be a possible initiating event in tauopathies (Kang et al, 2021). In fact, while our study was under review, an independent group also reported that the loss of function of either ANKLE2 or BANF1 resulted in increased tau aggregation, an outcome that was proposed to be caused by damage to the nuclear envelope, allowing for the leakage of nuclear components into the cytoplasm, which eventually triggers tau aggregation (Prissette et al, 2022). However, what is not clear is why cells with leaking nuclear components do not spontaneously develop tau aggregation, but rather require exogenous tau seeds to trigger tau aggregation (Prissette et al, 2022), as we also reported in this study. Apart from the role of BANF1 in nuclear envelope integrity, mutations in its gene can cause a human progeroid syndrome (Puente et al, 2011), and it appears that BANF1 is also crucial for restoring the capacity to repair oxidative lesions (Bolderson et al, 2019). Therefore, given that several studies have linked oxidative stress to tau pathology (Bartolome et al, 2022; Du et al, 2022), BANF1 could also have a role in decreasing the oxidative stress linked to tau aggregation. Unfortunately, we did not detect BANF1 in human brain samples by Western blotting, possibly because it is a very small protein of only 10 kD that could have been easily degraded during the postmortem interval of human tissue sampling, or alternatively, the aging brain may exhibit only low levels of BANF1.

Like tau, NUSAP1 is a microtubule-stabilizing protein that is found in the cytoplasm and nucleolus. It localizes to the mitotic spindle in dividing cells and causes mitotic arrest when overexpressed (Li et al, 2007). Furthermore, a potential epistatic genetic interaction has been reported between the SNP rs16971798 of NUSAP1 and the presence of tau filaments (Wang et al, 2020). The nucleolus is the site of ribosome biogenesis and rRNA processing (Koren et al, 2020), and nucleolar dysfunction has been reported in AD patients as a potential link to the development of tauopathies (Hernandez-Ortega et al, 2016; Maina et al, 2018a, 2018b; Nyhus et al, 2019). Our study is the first to report that a loss of function of NUSAP1 can facilitate tau aggregation and that the protein is down-regulated in AD brain samples, potentially leading to nucleolar dysfunction and thereby enhancing tauopathy.

Continuing with the theme of ribosomal dysfunction, we also identified EIF1AD that is required for the final steps of 40S ribosomal subunit assembly as shown in human cells (Ameismeier et al, 2020). To our knowledge, there is no report directly linking EIF1AD with tauopathies; however, EIF1AD has been linked to other brain diseases such as bipolar disorder (Stahl et al, 2019), which is associated with a significantly higher risk of dementia (Diniz et al, 2017; Mendez et al, 2020). We speculate that because of the role of EIF1AD in the formation of the ribosome, its down-regulation could cause ribosomal dysfunction and propitiate anomalous protein aggregation, given that translational impairment has been associated with tauopathies (Evans et al, 2019; Koren et al, 2020).

A limitation of our study is that tau biosensor cells are a non-neuronal cell line. However, to address this potential confound, we focused on genes with reported expression in neurons of the human brain to perform validations, with the aim of identifying conserved mechanisms between tau biosensor cells and neurons. This focus is reflected by our detection of validated targets in human brain samples from control and AD patients. Future studies with tau transgenic mice may increase our understanding of the role of the discovered regulators in the spreading and formation of tau pathology in brains. A recent report emphasized the inability of tau biosensor cells to form authentic paired helical filaments after seeded tau aggregation (Kaniyappan et al, 2020). Given that tau biosensor cells do not express full-length tau but rather a shorter version comprising the microtubule-binding domain tagged with fluorescent proteins, it is not unexpected that the induced tau aggregates are not formed exclusively by paired helical filaments but by other pathological conformations such as tau oligomers or short filaments. On the contrary, given that tau exists as six major isoforms in the human brain, one might even need to establish isoform-specific biosensor systems to fully capture all seeding-competent tau. It should also be noted that, in our study, we used tau biosensor cells as a sensitive assay that responds to exogenous aggregation-competent tau such as sarkosyl-insoluble tau and tau seeds within exosome-like EVs, with the goal of finding endogenous cellular regulators that control the tau seeding process. We did not aim to study or reproduce the structural conformation of the exogenous tau seeds.

### Concluding remarks

We have used CRISPRi-based functional genomics to reveal novel genetic players that predispose cells to tau aggregation. Intriguingly, at least three gene products, VPS18, NUSAP1, and EIF1AD, were found to be down-regulated in the AD brain, supporting their potential relevance in the emergence or propagation of tauopathy. Our data therefore raise the possibility that, for some of the genes revealed in our screen, (mutations or) gene variants may be identified that are associated with human tauopathies. Furthermore, all genes validated from our screen had an impact on seeded tau aggregation initiated by both vesicle-free and exosomal tau seeds, implying that therapeutically targeting our protein hits would not render neurons exposed to the attack of either free or membrane-bound tau but holistically cover both entry routes (Polanco & Götz, 2021), thereby representing a more efficacious and unifying treatment strategy for tauopathies.

## Materials and Methods

### Mouse strains and collection of brain tissue

Transgenic rTg4510 mice expressing human tau containing the P301L mutation that has been linked with familial frontotemporal dementia (Santacruz et al, 2005) were used at 6–12 mo of age for isolation of exosome-like EVs and sarkosyl-insoluble tau from dissected brains.

### Culture of tau biosensor cells and HEK293 Lenti-X cells

The “tau biosensor cells” are a monoclonal HEK293T cell line that stably expresses two fluorescently tagged forms of the microtubule-binding domain of tau bearing the P301S mutation, RD-CFP and RD-YFP, and were kindly provided by Dr. Marc Diamond (Holmes et al, 2014). BSKRAB cells were generated by transducing tau biosensor cells with lentiviral pLX_311-KRAB-dCas9 and used for CRISPRi genome-wide screening. BSKRAB-KD (individual knockdown) cells used in validation experiments were generated by transducing tau biosensor cells with lentiviral pLV hU6-sgRNA hUbC-dCas9-KRAB-T2a-Puro targeting each gene individually (Table S2). Lenti-X 293T cells (632180; Takara) were a subclone of the HEK293T cell line, which supports high levels of viral protein expression. All cells were grown in DMEM (11965092; Thermo Fisher Scientific) supplemented with 100 U/ml of penicillin-streptomycin (15140122; Thermo Fisher Scientific), 2 mM GlutaMAX (35050061; Thermo Fisher Scientific), and 10% FBS (SFBS-FR; Scientifix).

Table S2 Oligonucleotides encoding sgRNAs for individual knockdowns of hits.

### Plasmids and sgRNA cloning

The human Dolcetto CRISPR inhibition pooled library (#92385; Addgene), and the plasmids pLX_311-KRAB-dCas9 (#96918; Addgene), pLV hU6-sgRNA hUbC-dCas9-KRAB-T2a-Puro (#71236; Addgene), psPAX2 (#12260; Addgene), and pMD2.G (#12259; Addgene) were a kind gift from John Doench, David Root, Charles Gersbach, and Didier Trono to Addgene. For phenotypic validation, each sgRNA hit (Table S2) was individually cloned as annealed oligonucleotides into pLV hU6-sgRNA hUbC-dCas9-KRAB-T2a-Puro using Golden Gate cloning (Engler et al, 2008) with FastDigest Esp3I (FD0454; Thermo Fisher Scientific) and T4 DNA ligase (K1422; Thermo Fisher Scientific). All oligonucleotides were purchased from IDT, and the generated plasmids were corroborated by sequencing.

### Isolation of sarkosyl-insoluble tau from brains of tau transgenic mice

Biochemical isolation of sarkosyl-insoluble tau from brains of rTg4510 mice was performed as previously described (Julien et al, 2012). Briefly, one brain was homogenized in 3 ml of ice-cold 1× RIPA buffer (150 mM NaCl, 50 mM Tris–HCl, pH 7.4, 0.5% [wt/vol] sodium deoxycholate, 1.0% [vol/vol] Nonidet P-40, 5 mM EDTA, 50 mM NaF, and 200 mM NaVO_4_) containing 1× complete protease inhibitor cocktail (Roche) using a drill-driven Teflon Dounce homogenizer. Homogenate was centrifuged at 20,000*g* for 20 min, and the supernatant was mixed 1:1 with RIPA buffer with 2% sarkosyl (L9150; Sigma-Aldrich) and incubated for 1 h at room temperature with shaking. Insoluble tau was pelleted at 120,000*g* for 70 min, resuspended in 500 µl PBS (17-516Q; Lonza) with protease inhibitors, and sonicated with three 10-s pulses at 30% amplitude using a probe sonicator (Sonics Vibra-Cell). Sonicated sample was diluted with 3.5 ml PBS and concentrated to 500 µl by diafiltration using an Amicon Ultra-4 Centrifugal Filter Unit with 30 kD cutoff (UFC803024; Merck) to remove traces of sarkosyl. Protein content was quantified with a BCA protein assay kit (23227; Thermo Fisher Scientific).

### Isolation and purification of brain exosome-like EVs

Exosome-like EVs were isolated from the interstitial space of the mouse brain using a previously established protocol (Polanco et al, 2016, 2018a, 2021). In brief, each brain was chopped, and the cells were dissociated for 30 min at 37°C with 0.2% collagenase type III (LS004182; Worthington) in Hibernate-A medium (A1247501; Thermo Fisher Scientific), followed by gentle pipetting with a 10-ml pipette. A series of differential 4°C centrifugations at 300*g* for 10 min, 2,000*g* for 10 min, and 10,000*g* for 30 min was then performed to discard the pellets containing cells, membranes, and nanodebris, respectively. The supernatant from the 10,000*g* centrifugation step was passed through a 0.22-µm syringe filter (Millex-GP; Millipore) and ultracentrifuged at 120,000*g* for 70 min at 4°C to pellet the exosome-like EVs. Pellets from five mouse brains per genotype were pooled, washed with PBS, and ultracentrifuged. This pooled preparation of exosome-like EV pellets was resuspended in 2 ml of 0.95 M sucrose in 20 mM Hepes (15630080; Thermo Fisher Scientific), after which a sucrose step gradient (six 2 ml steps: 2.0, 1.65, 1.3, 0.95, 0.6, and 0.25 M on top) was used to purify the exosome-like EVs by centrifugation at 200,000*g* for 16 h at 4°C. Finally, the sucrose-purified exosome-like EVs floating in the interphase between 0.95 and 1.3 M sucrose were recovered, washed with 5 ml PBS, and ultracentrifuged again, and the exosome-like EV pellet was resuspended in 120 µl PBS containing 1× complete protease inhibitor cocktail (Roche) and 100 U/ml of penicillin-streptomycin (15140122; Thermo Fisher Scientific). Protein content was quantified with a BCA protein assay kit using a 15 µl aliquot of exosome-like EVs in PBS, which was mixed with 15 µl of 1× RIPA buffer (150 mM NaCl, 50 mM Tris–HCl, pH 7.4, 0.5% [wt/vol] sodium deoxycholate, 1.0% [vol/vol] Nonidet P-40, 1% [wt/vol] SDS, 5 mM EDTA, and 50 mM NaF) supplemented with protease inhibitors, and then homogenized in a water bath sonicator for 10 min.

### Fluorescent labeling of sarkosyl-insoluble tau and exosome-like EVs

Approximately 1 mg of sarkosyl-insoluble tau as 500 µl PBS solution at 2 mg/ml was fluorescently labeled on the N-terminus of protein aggregates using an Alexa Fluor 647 Protein Labeling Kit (A20173; Thermo Fisher Scientific) following the manufacturer’s instructions. For brain-derived exosome-like EVs, 600 µg protein equivalents of exosome-like EVs pelleted by ultracentrifugation were resuspended in 500 µl Diluent-C for membrane labeling (CGLDIL; Sigma-Aldrich) and then mixed 1:1 with 500 µl Diluent-C containing 2 µl CVC Far-Red Fluorescent Membrane Linker (MINCLARET; Sigma-Aldrich), labeling mixture that was incubated for 10 min at room temperature in the dark. Labeled exosome-like EVs were diluted with 3.5 ml PBS containing 1× complete protease inhibitor cocktail (Roche) and concentrated to 300 µl by diafiltration using an Amicon Ultra-4 Centrifugal Filter Unit with 30 kD cutoff (UFC803024; Merck) to remove the potentially unincorporated dye. The protein content of fluorescently labeled exosome-like EVs and sarkosyl tau was determined by a BCA protein assay as described above.

### Library production

Human CRISPRi sgRNA library Dolcetto Set A (Sanson et al, 2018) (#92385; Addgene) was transformed into electrocompetent Lucigen Endura *Escherichia coli* (60242-2; Lucigen) using program EC1 on MicroPulser Electroporator (1652100; Bio-Rad) following the manufacturer’s instructions. The electroporated bacteria were plated onto 10 × 15 cm LB agar dishes with 100 µg/ml ampicillin. After incubation for 16 h at 32°C, the bacteria were collected with a scraper in 5 ml PBS per dish, and plasmid DNA was extracted with the NucleoBond Xtra Midi kit (740410.50; Macherey-Nagel). The transformation efficiency was assessed by plating 1/10,000 of the reaction onto a 15-cm LB-agar plate with 100 µg/ml ampicillin.

### Production of lentiviral particles

Small-scale production of active lentiviral particles was performed with third-generation lentiviral transfer plasmids (500 ng each) mixed with 500 ng of a packaging DNA premix using psPAX2 and pMD2.G in a 2:1 ratio, which were transfected into Lenti-X 293T cells using TransIT-VirusGEN (MIR6700; MiRus) according to the manufacturer’s instructions for a 12-well plate. The transfection mixture was added to Lenti-X 293T cells cultured in DMEM containing 10% FBS. Lentivirus-containing conditioned medium was collected after 60 h, centrifuged at 1,000*g* for 5 min, and then filtered at 0.45 µm. Cells were transduced in DMEM supplemented with 10 mM Hepes and 8 µg/ml of Polybrene (H9268; Sigma-Aldrich) immediately before the conditioned medium being added.

A larger scale procedure was used for pooled CRISPRi library production in which Lenti-X 293T cells were seeded in 15-cm tissue culture dishes at a density of ∼10^5^ cells per cm^2^ overnight before transfection with the CRISPRi library (20 µg/dish), and packaging plasmids pMD2.G (5 µg/dish) and psPAX2 (25 µg/dish), using the transfection reagent TransIT-LT1 (152 µl/dish; MIR2300; MiRus). The DNA mixture was suspended in 6 ml of DMEM (11965092; Thermo Fisher Scientific). The solution was incubated at room temperature for 20 min, during which time the growth medium was changed on the Lenti-X 293T cells. After this incubation, the transfection mixture was added dropwise to Lenti-X 293T cells, and the plates were incubated at 37°C for 8 h. The transfection medium was then removed and replaced with DMEM containing 10% FBS supplemented with 0.5% BSA. Lentivirus-containing medium was collected 48 h later and centrifuged at 3,000*g* for 10 min at 4°C, and the supernatant was aliquoted and stored at −80°C. The virus titer was determined by serial dilution in HEK293T cells followed by puromycin selection (1 µg/ml) starting 48 h post-infection. The number of puromycin-resistant cells was used as a measure of the virus infectious units.

### Tau aggregation in tau biosensor cells transduced with a CRISPRi library

CRISPRi library cells were generated by transducing BSKRAB cells in four biological replicates at a low multiplicity of infection (∼0.5 MOI) with the Dolcetto lentiviral library, achieving an estimated representation of 1,000 cells per sgRNA per replicate. Transduced cells were selected with puromycin (1 µg/ml) starting 48 h post-infection. For genome-wide screens, 13 × 10^6^ CRISPRi library cells were seeded in T175 flasks overnight. The next day, the medium was aspirated in cells at ∼70% confluence, followed by treatment with 120 µg sarkosyl-insoluble tau (vesicle-free tau seeds) resuspended in 35 ml DMEM supplemented with penicillin-streptomycin and GlutaMAX as above, but using 5% exosome-depleted fetal bovine serum (edFBS) prepared by centrifugation of FBS at 120,000*g* for 18 h, followed by filter sterilization of the supernatant. CRISPRi library cells strongly developed tau inclusions by 48 h, at which time the cells were analyzed by FRET flow cytometry as described below.

### FRET flow cytometry

Tau aggregation between RD-CFP and RD-YFP was visualized and quantified by FRET flow cytometry as previously described (Holmes et al, 2014; Polanco et al, 2016, 2021). In brief, CRISPRi library cells in T175 flasks used for the screens were harvested with 3 ml 0.25% trypsin–EDTA (25200056; Thermo Fisher Scientific) at 37°C for 5 min, mixed with five volumes of culture medium, and centrifuged at 300*g* for 5 min. The supernatant was then aspirated, after which the cell pellet was washed with PBS, and then resuspended in ice-cold FACS buffer (PBS containing 30 mM Hepes, 0.5 mM EDTA, and 0.2% BSA). FRET flow cytometry was subsequently performed using a FACSAria cell sorter (Becton Dickinson), where cells were excited by a 405-nm laser (Coherent Inc.), and the emitted fluorescence was captured with filters for 485/22 nm to detect CFP and 530/30 nm to detect FRET, gating the cells as outlined previously (Polanco et al, 2016, 2021). For genomic screens, all the FRET-positive and FRET-negative cells from T175 flasks were sorted and collected independently. For validation assays in 96-well plates, cells were washed with PBS before being dissociated with 40 µl 0.25% trypsin–EDTA without phenol red (15400054; Thermo Fisher Scientific) and then mixed in the well with 160 µl DTI FACS buffer prepared with Defined Trypsin Inhibitor (R007100; Thermo Fisher Scientific) supplemented with 30 mM Hepes, 0.5 mM EDTA, and 0.2% BSA. FRET flow cytometry was performed as above, analyzing 40,000 cells per triplicate in each experiment. FRET data were quantified as the integrated FRET signal, calculated by multiplying the percentage of FRET-positive cells in the sample by their respective mean 530-nm fluorescence intensity generated by FRET.

### Validation of hits in individual knockdowns

For validation experiments, tau biosensor cells were transduced in 12-well plates with lentiviruses targeting each hit individually, by cloning each sgRNA into pLV hU6-sgRNA hUbC-dCas9-KRAB-T2a-Puro, and using non-targeting sgRNAs as a control (Table S2). Each well contained an individual knockdown cell line (BSKRAB-KD), and these cells were split in DMEM culture medium with puromycin (1 µg/ml) plus 10% edFBS after 72-h transduction. Individual BSKRAB-KD cells from each 12-well plate were plated on 96-well plates in triplicate at a density of 20,000 cells per well overnight using 100 µl of puromycin-containing medium. The next day, 50 µl of culture medium was removed and replaced with either 400 ng sarkosyl-insoluble tau or 3 µg protein equivalents of exosome-like EVs prepared in 50 µl fresh culture medium, incubating the treated BSKRAB-KD cells for a further 72 h before FRET flow cytometry analysis as described above. Cells were topped up with 100 µl fresh medium at 48 h to avoid acidification of the culture medium. In parallel, 600,000 BSKRAB-KD cells per individual knockdown were seeded in six-well plates and grown for 72 h, to prepare whole-cell lysates to corroborate knockdowns by Western blots.

### Uptake quantification of exosome-like EVs and vesicle-free tau seeds

To measure levels of tau seed uptake, individual knockdown BSKRAB-KD cells were plated on 96-well plates at a density of 50,000 cells per well using 100 µl DMEM plus edFBS. 48 h later, 50 µl of culture medium was removed and replaced with either 400 ng sarkosyl-insoluble tau labeled with Alexa Fluor 647 or 2 µg protein equivalents of exosome-like EVs labeled with far-red CVC prepared in 50 µl fresh culture medium with edFBS. These cells were incubated at 37°C for 60 min; after which the medium was aspirated, the cells were washed with PBS, dissociated with 40 µl of 0.25% trypsin–EDTA without phenol red (15400054; Thermo Fisher Scientific) at 37°C for 10 min, and finally mixed with ice-cold 160 µl Defined Trypsin Inhibitor (R007100; Thermo Fisher Scientific). To remove potential traces of non-internalized labeled tau seeds, trypsin-dissociated cells on 96-well plates were centrifuged at 1,000*g* for 5 min at 4°C using a swinging-bucket rotor for microplates (S6096; Beckman Coulter) in an Allegra X-30R centrifuge (Beckman Coulter). The supernatant was aspirated after centrifugation, and cell pellets were resuspended in 200 µl ice-cold DTI FACS buffer (formulation above) before flow cytometry using a BD LSR II instrument. Tau uptake was quantified by measuring the mean fluorescence intensity in far-red–positive cells, analyzing 40,000 cells per triplicate in each experiment. Uptake inhibition was carried out either in the cold (4°C) or by incubation with the dynamin inhibitor, Dyngo-4a (ab120689; Abcam). Dyngo-4a stocks were prepared at 30 mM in DMSO and used at a concentration of 60 μM. Both the inhibitor and vehicle (DMSO) were prepared in medium, and 50 μl of the culture medium was replaced 30 min before seeding. At the time of seeding, the culture medium was removed and replaced with the one prepared with labeled seeds together with the inhibitor or vehicle. For the cold treatments, the labeled seeds were prepared and incubated at 4°C an hour before seeding. 50 μl of culture medium was removed and replaced with the precooled seeds and subsequently incubated at 4°C. Downstream processes were as for the treatment conditions.

### Genomic DNA preparation and NGS

Genomic DNA from FRET-positive and FRET-negative cells was isolated using commercial kits (13323 and 13343; QIAGEN). We amplified sgRNA cassettes from gDNA (2.5 µg) using OneTaq DNA Polymerase (#M0480; New England Biolabs) and LG.Lib.ampl1.F and LG.Lib.ampl1.R primers in a 50 µl reaction. Illumina sequencing primer-binding sites were then added by PCR amplification of sgRNA amplicons with primer mix LG.LibAmpl.WSstag.mix and LG.gRNA.Ampl.NGS.R. Lastly, Illumina indices and adapters for sample multiplexing were added by PCR amplification with Illumina_indX_F and Illumina_indX_R primers. The last two PCR rounds were performed using NEBNext Ultra II Q5 Master Mix (#M0544; New England Biolabs). Samples were purified using AMPure XP beads (#A63880; Beckman Coulter). The library was sequenced with coverage of 200 reads per sgRNA using the PE100 protocol on NextSeq 500 Illumina Sequencer (with 10% PhiX spike-in). Samples were demultiplexed, and spacers were counted using the count_spacers.py script (Joung et al, 2017). Positively selected genes were identified using the MAGeCK tool (Li et al, 2014). The over-representation and gene set enrichment analyses for GO-BP (biological process) and GO-CC (cellular component) terms were performed using the clusterProfiler R package (Yu et al, 2012). Primer sequences and detailed reaction setups are listed in Table S3.

Table S3 Primer sequences and detailed reaction setups for next-generation sequencing.

### Bioinformatic analysis

Samples were demultiplexed using Illumina bcl2fastq to generate FASTQ files. Individual sgRNA counts were extracted using the count_spacers.py script (Joung et al, 2017). Positively selected genes were identified using the MAGeCK tool (Li et al, 2014) and DESeq2 (Wald) (Love et al, 2014) using simplified routines provided by the DEBRA R package (Akimov et al, 2020). The over-representation and gene set enrichment analyses for GO-BP (biological process) and GO-CC (cellular component) terms were performed with the clusterProfiler R package (Yu et al, 2012) using the first 200 genes with the following parameters pAdjustMethod = “BH,” pvalueCutoff = 0.25, and qvalueCutoff = 0.25.

### Brain and cell lysates

HEK293T cells grown in six-well plates were used to prepare whole-cell lysates using a pellet of trypsin-dissociated cells that was homogenized in 200 µl RIPA buffer (150 mM NaCl, 50 mM Tris–HCl, pH 7.4, 0.5% [wt/vol] sodium deoxycholate, 1.0% [vol/vol] Nonidet P-40, 1% [wt/vol] SDS, 5 mM EDTA, and 50 mM NaF) with protease inhibitors using a probe sonicator (Sonics Vibra-Cell) for 20 s at 30% amplitude. Sonicated lysates were left solubilizing on ice for 1 h, then centrifuged at 20,000*g* for 20 min, using the supernatant for protein quantification with a BCA protein assay. Similarly, human brain lysates from postmortem AD patients were prepared with 25 mg of superior frontal cortex tissue homogenized in 500 µl RIPA buffer supplemented with protease and phosphatase inhibitors, disrupting the tissue with 20 strokes of a drill-driven Teflon Dounce homogenizer, solubilizing the lysates on ice for 30 min, and then centrifuging at 20,000*g* for 20 min, taking the supernatant for protein quantification.

### Western blot analysis

Criterion TGX 4–15% (5671084; Bio-Rad) and Mini-Protean TGX (4561083; Bio-Rad) precast gels were used to separate 20–40 µg of total protein from lysates, which were then transferred onto Immuno-Blot low-fluorescence PVDF membranes (1704275; Bio-Rad) using the Trans-Blot Turbo transfer system (Bio-Rad). Membranes were blocked in Odyssey Blocking Buffer (LI-COR) for 1 h at room temperature and then incubated overnight at 4°C with primary antibodies prepared in Odyssey Blocking Buffer with 0.01% Tween-20. Membranes were washed with Tris-buffered saline/0.1% Tween-20 (TBST) three times for 10 min at RT. Then, IRDye secondary antibodies (LI-COR) were added and diluted 1:10,000 in a 1:1 mixture of Odyssey Blocking Buffer with TBST for 1 h at room temperature. Finally, membranes were again washed three times in TBST, and the fluorescence signals were recorded using an Odyssey FC imaging system (LI-COR). Analysis and protein quantification were performed using the Image Studio software (LI-COR). The following antibodies were used: anti-human ANKLE2 rabbit polyclonal (1:1,000; A302-965A; Thermo Fisher Scientific), anti-human EIF1AD rabbit polyclonal (1:1,000; 20528-1-AP; Thermo Fisher Scientific), anti-BANF1/BAF rabbit monoclonal (1:1,000; ab129184; Abcam), anti-human NUSAP1 rabbit polyclonal (1:500; ab137230; Abcam), anti-human VPS18 rabbit monoclonal (1:1,000; ab178689; Abcam), anti-human Tau-13 mouse monoclonal (1:1,000; MMS-520R; Covance), anti-tau phospho-Ser422 rabbit polyclonal (1:1,000; GTX86147; GeneTex), and the normalizers anti-GAPDH mouse monoclonal (1:2,000; MAB374; Millipore) and anti-GAPDH rabbit polyclonal (1:2,000; 10494-1-AP; Proteintech).

### Statistical analysis

To determine the statistical significance of differences in quantification levels in validation experiments, *P*-values were determined either from a two-tailed unpaired *t* test with Welch’s correction or from one-way ANOVA with a 95% confidence interval and Dunnett’s test to correct for multiple comparisons, and calculated with GraphPad Prism v9.3 for Windows (GraphPad Software Inc.).

### Ethics approval and consent to participate

Animal experimentation was approved by the Animal Ethics Committee of the University of Queensland (approval nos. QBI/505/17/NHMRC and QBI/554/17/NHMRC). Human brain samples (superior frontal cortex) were received from the New South Wales Brain Tissue Resource Centre at the University of Sydney and Sydney Brain Bank at Neuroscience Research Australia, which are supported by the University of New South Wales, Neuroscience Research Australia, and the Schizophrenia Research Institute. The research reported in this publication was supported by the National Institute of Alcohol Abuse and Alcoholism of the National Institutes of Health under Award Number R28AA012725. The content is solely the responsibility of the authors and does not necessarily represent the official views of the National Institutes of Health.

## Supplementary Material

Reviewer comments
